# Does Stapling Platform Influence Robotic Sleeve Gastrectomy Postoperative Outcomes?

**DOI:** 10.1007/s11695-025-07855-z

**Published:** 2025-04-14

**Authors:** Lee Ying, Rachael Rutledge, Samuel Butensky, Daniel Farinas Lugo, John Morton, Forrest Ringold

**Affiliations:** 1 Yale New Haven Hospital, New Haven, USA; 2 Mobile Infirmary, Mobile, USA; 3 Yale School of Medicine, New Haven, USA

**Keywords:** Stapling, Obesity, Sleeve gastrectomy, Outcomes

## Abstract

**Background:**

Laparoscopic sleeve gastrectomy (LSG) accounts for the majority of weight loss surgeries worldwide. Although the overall side effect profile is low, the rate of *de novo* gastroesophageal reflux disease (GERD) ranges between 2.1 and 49%. Our study compares postoperative outcomes using a single-fire stapler versus a multiple-fire linear stapler, with a focus on postoperative *de novo* GERD.

**Methods:**

This was a retrospective single-surgeon study with data from 257 patients who underwent consecutive robotic-assisted sleeve gastrectomy between 2016 and 2023 with either multiple fires of a linear stapler (*n* = 201) or a single-fire linear stapler (*n* = 56). Patient demographics and postoperative outcomes, including 30-day complications, 1-year weight loss, and 1-year postoperative reflux, were analyzed.

**Results:**

Patients in the single-fire group were noted to have a significantly lower rate of postoperative reflux (7.1% vs. 26.4%) and a decreased incidence of *de novo* reflux (1.8% vs. 10.9%). Additionally, the single-fire group had a shorter average length of stay (2.0 days vs. 2.2 days, *p* = 0.04). Multivariable analysis demonstrated that single-fire stapler use increased the likelihood of not developing postoperative GERD (odds ratio: 8.4, 95% confidence interval: 2.8–32.5). There was no significant difference in operative time (multiple-fire group: 81.4 min, single-fire group: 90.1 min, *p* = 0.5) or 1-year percent total weight loss (multiple-fire: 22.4% ± 0.7%, single-fire: 22.0% ± 1.7%, *p* = 0.8).

**Conclusions:**

Single-fire stapler use may enhance postoperative outcomes in LSG by reducing rates of *de novo* GERD without impacting weight loss. There was no significant difference in operative time, and postoperative length of stay may be decreased.

## Introduction

The global prevalence of obesity continues to rise, and recent census data estimates that 1 in 8 persons currently lives with obesity [1]. Bariatric and metabolic surgery remains the most effective and definitive treatment, and laparoscopic sleeve gastrectomy (LSG) has become the most frequently performed weight loss surgery worldwide, accounting for more than 50% of all surgeries annually [2]. Despite its prevalence, there is significant variability in postoperative outcomes after LSG, particularly in the incidence of *de novo* postoperative gastroesophageal reflux disease (GERD). In the literature, the prevalence of GERD after LSG ranges from 2.1 to 49%, with variability likely due to heterogeneity in patient anatomy and preoperative body mass index (BMI), method of assessing GERD, and operative technique [3].

The etiology of postoperative GERD after LSG is multifactorial, but postoperative alterations in anatomy are believed to be contributory [4]. In a standard LSG, dissection carried along the greater curvature may disrupt the muscle fibers at the angle of His, an anatomical feature that functions as an anti-reflux valve and is protective against GERD in its unperturbed state [5]. Volume and pressure assessments performed by Yehoshua et al. have demonstrated that decreased gastric compliance and volume after creation of the sleeve configuration results in increased gastric pressure and reduced distensibility, conditions that facilitate GERD [6]. Finally, improperly placed staple fires during sleeve creation can cause narrowing and torsion of the sleeve, which has also been linked to GERD development.

Postoperative GERD is a significant source of morbidity after LSG, and not only negatively impacts patient satisfaction and quality of life as a source of nausea and vomiting, but can lead to extended hospital length of stay (LOS) and contributes to readmissions and reoperations [7]. Severe and refractory GERD is also a top contributor for conversion to Roux-en-Y gastric bypass. Given the importance of the sleeve configuration in the development of GERD, reducing variability in postoperative anatomy may reduce its incidence. The Titan® SGS stapler is a non-articulating, 23 cm length, powered stapling device that was developed to improve consistency in LSG by utilizing a single-fire staple mechanism [8]. A traditional sleeve gastrectomy requires multiple firings of a linear stapler to traverse the length of the gastric greater curvature, which results in greater variability and potential for misplaced fires. The proposed benefits of the single-fire stapler include decreased operative time, removal of junctions in the staple line, and elimination of angulation between staple loads [8]. Additionally, a single-fire stapler eliminates overlapping staple lines, which carries a Food and Drug Administration (FDA) warning for increased leak risk [9]. In this retrospective analysis, we aim to determine if use of a single-fire stapler is associated with improved postoperative outcomes after LSG, including decreased length of stay and reduced GERD symptomology. This study reflects a single-surgeon experience with robotic-assisted creation of a sleeve gastrectomy using multiple fires of a traditional linear stapler (‘multiple-fire group’) versus a single fire of a non-articulating linear stapler (‘single-fire group’).

## Methods

### Operative Details

All operations were performed by a single surgeon. In both the single-fire and multiple-fire groups, access was obtained using a Veress needle. An 8 mm trocar was placed 17 cm inferior to the xiphoid process and slightly to the left of midline. Four additional trocars were then placed under direct laparoscopic vision. A ratcheted toothed 5 mm grasper was inserted via a small sub-xiphoid incision and used to retract the liver anteriorly and expose the esophageal hiatus. The patient was then placed in reverse Trendelenburg, and the robotic arms were docked. The initial dissection was performed by transecting the vasculature along the greater curvature of the stomach, starting approximately 5 cm proximal to the pylorus and carried proximally until the left crus of the diaphragm was exposed. A 40 French VISIGI® bougie (Boehringer Laboratories, LLC) was passed by the anesthetist along the lesser curve of the stomach to the antrum. The VISIGI® bougie was then placed on suction.

In the single-fire group, a 19 mm trocar was placed 23 cm inferior to the xiphoid process along a tangential line near the midclavicular plane. Three anatomical sites were located and marked internally with a marker: 1 cm lateral to the gastroesophageal junction, 3 cm lateral to the angularis incisura, and 5 cm proximal to the pylorus along the greater curvature. The single-fire stapler was opened by a Registered Nurse First Assist (RNFA) and the stomach was placed into the stapling device and aligned with the first mark within the stapler near its hinge by the console surgeon. The stapler was then partially closed. The second mark was then placed within the stapler and partially closed the stapler again. Finally, the third mark was placed within the stapler and the device was completely closed. The sleeve gastrectomy was then created by firing the stapler as a single firing, and the specimen was removed through the 19 mm trocar. In the multiple-fire group, the sleeve gastrectomy was performed using either two black and three purple 60 mm cartridges (Tri-Staple™, Medtronic), or two green and three blue 60 mm cartridges (SureForm, Intuitive). A 40 French bougie was used to size all sleeves.

In all cases, if a depression was noted in the crura, the entire esophageal hiatus was explored, and a hiatal hernia repair was performed after achieving a complete circumferential dissection around the esophagus and obtaining an intra-abdominal esophagus length of at least 3 cm. The crura were re-approximated over a 40 French bougie using a running nonabsorbable knotless suture with woven polytetrafluoroethylene pledgets to bolster the cruroplasty. The posterior gastroesphageal junction was affixed to the median arcuate ligament with permanent suture. 12 and 19 mm trocar sites were closed at the fascia level.

### Data Collection

This was a single-surgeon retrospective study of consecutive patients over a period of 5 years. Data for the multiple-fire cohort was collected from September 2018 until October 2021, at which point, the surgeon transitioned to exclusive use of the single-fire stapler until October 2023. A single patient underwent surgery with a multiple-fire stapler in the month after the transition. All data was retrospectively collected in a HIPPA-compliant excel spreadsheet. Preoperative variables were collected from the patient history and physical examination. GERD symptoms and anti-reflux medication use were assessed using the same standardized survey administered preoperatively, and then during regular postoperative follow-up intervals at 2 weeks, 1 month, 3 months, 6 months, 1 year, and then annually thereafter. The presence of GERD was diagnosed clinically by querying for typical symptoms, including reflux, heartburn, and regurgitation. At each visit, patients were also asked about current reflux medication use, including proton pump inhibitors, histamine- 2 blockers, and calcium-based antacids. Data regarding GERD were independently obtained by a nurse practitioner.

### Statistical Analysis

Results are presented as mean ± standard error of the mean. Differences between two means were calculated using a Student’s *T*-test. Proportions were compared using a chi-square test, or Fisher’s exact test. Odds ratios were calculated using multiple logistic regression analysis. All significance levels were set to *p* < 0.05. All statistical tests were performed on Prism 10 (GraphPad).

## Results

In total, there were 201 patients in the multiple-fire group, and 56 patients in the single-fire group. The average age of patients was 44.5 years in the multiple-fire group and 43.7 years in the single-fire group (*p* = 0.4). 79.1% of patients in the multiple-fire group were female, and 87.5% of patients in the single-fire group were female (*p* = 0.1). Preoperatively, the average BMI of patients in the single-fire group (47.9 ± 0.9) was significantly higher than that in the multiple-fire group (44.2 ± 0.5, *p* = 0.0004). Postoperatively, the average BMI of the single-fire group (36.5 ± 1.1) was not significantly higher than that in the multiple-fire group (34.4 ± 0.5, *p* = 0.0502). There was also no significant difference in the 1-year total body weight loss percent (multiple-fire: 22.4% ± 0.7%, single-fire: 22.0% ± 1.7%, *p* = 0.8). There was no significant difference in the distribution of Black, White, or Hispanic patients (Table 1). In terms of co-morbidities, 57.7% of patients in the multiple-fire group and 55.4% of patients in the single-fire group had pre-existing hypertension (*p* = 0.4). There was also no significant difference in the percentage of patients with diabetes (multiple-fire: 23.9%, single-fire: 30.4%; *p* = 0.4), or with a hiatal hernia (multiple-fire: 40.8%, single-fire: 42.9%, *p* = 0.9).

**Table 1:** Demographics

	Multiple-fire (*n* = 201)	Single-fire (*n* = 56)	*p*-value
Age (years)	44.5 ± 0.8	43.7 ± 1.1	0.4
Percent female	159 (79.1%)	49 (87.5%)	0.1
BMI (kg/m^2^)			
Preoperative	44.2 ± 0.5	47.9 ± 0.9	0.0004
1-year	34.4 ± 0.5	36.5 ± 1.1	0.0502
Weight (kg)			
Preoperative	126.2 ± 1.8	127.8 ± 3.6	0.7
1-year	97.6 ± 1.6	98.7 ± 2.9	0.7
1-year %total weight loss	22.4% ± 0.7%	22.0% ± 1.7%	0.8
Ethnicity			
Black	55 (27.4%)	27 (48.2%)	0.1
White	144 (71.6%)	29 (51.8%)	
Hispanic	27 (13.4%)	0	
Co-morbidities			
Hypertension	116 (57.7%)	31 (55.4%)	0.8
Diabetes	153 (23.9%)	39 (30.4%)	0.4
Hiatal hernia (repaired at index operation)	82 (40.8%)	24 (42.9%)	0.9
Intraoperative			
Operative time (minutes)	81.4 ± 1.7	90.1 ± 3.9	0.3
Length of stay (days)	2.2 ± 0.04	2.0 ± 0.03	0.03
Concomitant	21 (10.4%)	4 (7.1%)	0.6
Cholecystectomy			
Complications			
Leak	0	0	n/a
30-day readmission	8 (4.0%)	0 (0%)	0.2
Reoperation	0 (0%)	0 (0%)	n/a
Surgical site infection	0 (0%)	0 (0%)	n/a
Preoperative reflux	53 (26.4%)	17 (30.4%)	0.6
1-year postoperative reflux	53 (26.4%)	4 (7.1%)	0.002

The operative time for patients in the multiple-fire group was 81.4 min, which was shorter than the 90.1 min in the single-fire group, although the difference was not statistically significant (*p* = 0.5). However, patients in the single-fire group had a significantly shorter length of stay (2.0 ± 0.03 days) compared to patients in the multiple-fire group (2.2 ± 0.04, *p* = 0.04). There was no significant difference in the proportion of patients who underwent a concomitant cholecystectomy (multiple-fire: 10.4%, single-fire: 7.1%; *p* = 0.6). None of the patients in either group had a postoperative leak or staple line bleed. 4% of patients in the multiple-fire group had a 30-day readmission. None of the patients in the single-fire group had any 30-day readmissions, reoperations, or surgical site infections.

**Fig. 1:**
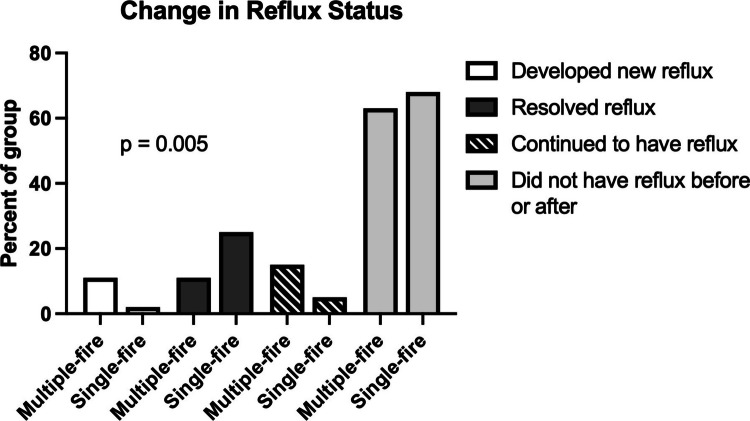
Change in reflux status after surgery with multiple-fire or single-fire stapler use. There was a significant difference in the overall proportion of patients who developed new reflux, continued to have reflux, had resolution of reflux after surgery, or continued to be free of reflux after surgery

Prior to surgery, 26.4% of patients in the multiple-fire group and 30.4% of patients in the single-fire group had reflux requiring medications (*p* = 0.6). Postoperatively, 7.1% of patients in the single-fire group had reflux, compared to 26.4% in the multiple-fire group (*p* = 0.002). We further characterized changes in reflux status by analyzing the proportion of patients who developed de novo reflux (postoperative reflux without preoperative reflux), had resolution of their reflux (preoperative reflux without postoperative reflux), continued to have reflux (preoperative and postoperative reflux), or continued to not have reflux (no reflux preoperatively or postoperatively), and found significant differences in the overall proportions (*p* = 0.005, Fig. 1). Most patients in both groups did not have reflux before or after surgery (multiple-fire: 62.7%; single-fire: 67.9%). However, in the multiple-fire group, 10.9% of patients developed new reflux symptoms after surgery, compared to only 1.8% in the single-fire group. Additionally, 15.4% of patients in the multiple-fire group had reflux before and after surgery, compared to only 5.4% in the single-fire group. Finally, 25% of patients in the single-fire group reported resolution of their reflux after surgery, compared to 10.9% in the multiple-fire group.

**Fig. 2:**
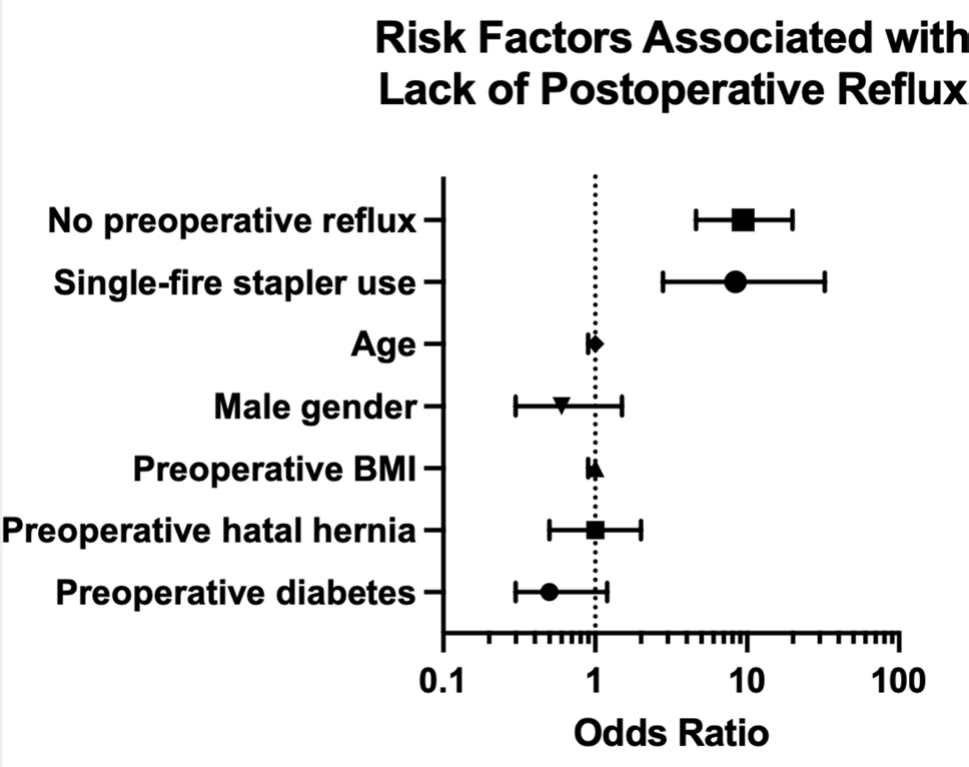
Multiple logistic regression analysis was performed including the following variables: age, BMI, presence of preoperative diabetes, presence of a preoperative hiatal hernia, absence of preoperative reflux, and use a single-fire stapler (odds ratio [95% confidence interval]). Absence of preoperative reflux (9.4 [4.6–19.9]) and use of a single-fire stapler (8.4 [2.8–32.5]) were the strongest predictors for absence of postoperative reflux

To better understand the factors associated with improved 1-year postoperative reflux, we performed a multiple logistic regression analysis including the following variables: age, BMI, presence of preoperative diabetes, presence of a preoperative hiatal hernia, absence of preoperative reflux, and use a single-fire stapler (odds ratio [95% confidence interval]). Absence of preoperative reflux (9.4 [4.6–19.9]) and use of a single-fire stapler (8.4 [2.8–32.5]) were the strongest predictors for the absence of postoperative reflux (Fig. 2).

## Discussion

Bariatric and metabolic surgery remains the most effective and durable treatment for obesity, and laparoscopic sleeve gastrectomy (LSG) achieves excellent weight loss outcomes with a minimal side effect profile. Despite its popularity, there is significant variability in the development of de novo postoperative gastroesophageal reflux disease, and variations in staple placement during sleeve creation may be contributory [10]. Single-fire staplers may reduce variability in postoperative anatomy, and thereby reduce the incidence of postoperative GERD [11]. This single-surgeon study with 1-year follow-up included 257 patients and compared robotic-assisted sleeve gastrectomy creation with multiple fires of an articulating linear stapler to a single-fire of a non-articulating linear stapler. Our results suggests that single-fire stapler use is highly associated with absence of postoperative reflux (OR [95% CI]: 8.4 [2.8–32.5]).

Overall, the two groups were well balanced, and there was no significant difference in the average age, gender distribution, reported ethnicities, or preoperative co-morbidities. Interestingly, the patients in the single-fire group had a significantly higher average preoperative BMI and continued to have a small but significantly higher 1-year postoperative BMI. Even though high BMI is associated with reflux [12], the proportion of patients with 1-year postoperative reflux was considerably lower in the single-fire group. Importantly, there was no difference in 1-year total weight loss percent. All patients were followed for 1 year, suggesting that these results are not transient changes in the immediate postoperative period. As this was a single-surgeon study, there were also no significant differences in intraoperative practices between the two groups which were contemporaneous. Importantly, there was no difference in the proportion of patients with preoperative hiatal hernias, and it is the standard practice of this surgeon to repair all hiatal hernias that are discovered incidentally during surgery. All surgeries were done using a robotic platform, which allows for better evaluation of the impact of a single-fire stapler. No postoperative leaks, reoperations, or surgical site infections were reported in either group.

Our primary outcome was the presence or absence of postoperative reflux at 1 year after surgery. The proportion of patients who reported 1-year reflux was significantly lower in the single-fire group (multiple-fire: 26.4%, single-fire: 7.1%, *p* = 0.002), even though there was a higher but statistically non-significant proportion of patients in this group who reported preoperative reflux (multiple-fire: 26.4%, single-fire: 30.4%, *p* = 0.6). When we further analyzed patterns of change in reflux status, we found that a lower proportion of patients in the single-fire stapler group developed de novo reflux (multiple-fire: 10.9%, single-fire: 1.8%), and a higher proportion reported resolution of their reflux after surgery (multiple-fire: 10.9%, single-fire: 25%). One possible explanation for these findings is that using a single-fire stapler results in less kinking along the sleeve staple line, which prevents inadvertent narrowing. Interestingly, patients in the single-fire group did have a small but significantly shorter length of stay (2.0 ± 0.03 days) compared to patients in the multiple-fire group (2.2 ± 0.04, *p* = 0.04). While many factors influence length of stay, including postoperative complications, poor pain control, or social barriers to discharge, in our experience, a common cause for delayed discharge is intolerance of oral intake [13]. We hypothesize that patients may also have less postoperative nausea with single-fire stapler use, as it creates a uniform gastric sleeve with less hour-glassing and fewer angulations (colloquially referred to as ‘zigzags’ or ‘dog-ears’). The mechanisms underlying decreased 1-year reflux may also contribute to improved early oral intake, but future studies focused on assessing postoperative nausea after single-fire stapler will be required to better characterize these findings.

Our study does have some inherent limitations, including reliance on self-reported reflux symptoms corroborated with PPI use as a surrogate marker for GERD. This is an indirect measurement of GERD, and some studies have suggested that patients may continue PPI therapy even in the absence of clinical reflux due to concerns about symptom recurrence [14]. Empiric trials of proton pump inhibitor (PPI) therapy are often used to simultaneously diagnose and treat GERD, however, 35% of patients with no objective evidence of GERD report symptomatic relief from PPI therapy [15]. It is the opinion of these authors that surveying patients on the presence of GERD symptoms with concurrent anti-reflux medication use is a practical method for standardizing the assessment of improvements in GERD, as quantitative tests are expensive, invasive, and impractical to serially obtain, especially in asymptomatic patients.

It must be noted that a Roux-en-Y gastric bypass is the preferred operation in patients with concomitant reflux. During preoperative counseling, it is our practice to explain that gastric bypass tends to offer better reflux control compared to sleeve gastrectomy. However, many patients are averse to gastric bypass due to its associated complications and elect to undergo a sleeve gastrectomy despite the risks of de novo or worsening reflux. Our finding of a significant decrease in the rate of reported reflux in the single-fire group provides a compelling argument for the use of single-fire staplers in this patient population and suggests the need for future studies involving formal postoperative testing for GERD resolution, including endoscopy or combined pH-impedance monitoring to measure acid exposure time and analyze reflux-symptom association profiles.

## Conclusion

Laparoscopic sleeve gastrectomy is a highly effective treatment for obesity, but *de novo* postoperative gastroesophageal reflux disease remains a significant challenge. Our study suggests that use of a single-fire stapler is associated with a significantly lower incidence of GERD compared to traditional multiple-fire staplers 1 year postoperatively. Additionally, patients in the single-fire cohort had a lower rate of developing *de novo* GERD and a higher rate of GERD resolution. Despite no significant difference in operative time, the single-fire group had a shorter length of stay, which may reflect improved postoperative dietary intake. These findings suggest that the single-fire stapler may offer superior postoperative outcomes by reducing the risk of GERD after LSG, highlighting its potential clinical benefits in bariatric surgery.

## Data Availability

No datasets were generated or analysed during the current study.
